# HIV-Positive Patient With Non-Percutaneous Coronary Intervention (PCI)-Amenable Left Coronary Artery Aneurysms Presenting With ST-Elevation Myocardial Infarction (STEMI)

**DOI:** 10.7759/cureus.36703

**Published:** 2023-03-26

**Authors:** Raheel M Khan, Usman S Najam, Dany A Cheikh Debs, Jermaine Myers, Susan Graham

**Affiliations:** 1 Internal Medicine, University at Buffalo, Buffalo, USA; 2 Cardiology, University at Buffalo, Buffalo, USA

**Keywords:** coronary artery bypass grafting (cabg), st-elevation myocardial infarction (stemi), coronary artery aneurysms, hiv-positive, coronary artery angiogram

## Abstract

Human immunodeficiency virus (HIV) was first reported in the early 1980s and a once untreatable and fatal disease has since allowed individuals to live healthy lives with the advent of novel antiviral medications. While the life expectancy of an HIV-positive individual has dramatically increased, a myriad of HIV-related complications such as pneumocystis pneumonia, candidiasis, renal disease, anxiety/depression, and cardiovascular disease have dramatically decreased. However, these patients are still prone to complex medical problems. In this case report, we aim to highlight a rare, complicated case of an HIV-positive patient with coronary artery aneurysms complicated by an ST-elevation myocardial infarction (STEMI).

## Introduction

Human immunodeficiency virus (HIV) was first reported in the 1980s, and given the unexpected emergence and the lack of adequate antivirals during that time, HIV infection led to significant comorbidities and death. With time, a wide array of antiretroviral medications are now available, with the first HIV antiviral medication being azidothymidine (AZT), a nucleoside reverse transcriptase inhibitor (NRTI), which blocks RNA-dependent DNA polymerase preventing DNA replication via reverse transcriptase [1-3]. With the vast antiviral medications on the market, an HIV-positive individual's life expectancy has dramatically increased, and the myriad of HIV-related cardiac, pulmonary, infectious, renal, and psychiatric complications have dramatically decreased [2-4]. Due to the reduction in AIDS-related mortality with potent antiretrovirals, non-HIV-related mortality including cardiovascular mortality has become increasingly relevant. It is well known that HIV-positive patients have accelerated atherosclerosis due to multiple factors including a predisposition to relapsing/remitting inflammation, dyslipidemia, insulin resistance, and medication side effects. These associations are well documented in large vessels such as cerebral, aortic, and carotid arteries; however, the literature on small vessel injury is lacking.

A systematic review and meta-analysis reported that the relationship between HIV-positive patients and coronary artery aneurysms is underreported as there is only a 1.5-4.9% incidence of sporadic cases published thus far. HIV is a pro-inflammatory, pro-thrombotic, and dyslipidemic state as evidenced by low CD4/high CD8, low protein S, and low apolipoprotein B/elevated LDL-cholesterols, respectively. These in turn lead to inflammation and more importantly myocardial infarctions (MI) [3-6]. This is why HIV infection is considered a risk enhancer in the primary prevention cholesterol guidelines according to the American College of Cardiology (ACC) [7-8]. Oftentimes, patient-related characteristics or co-morbidities make decision-making difficult, especially when related to acute coronary syndromes. We describe a complicated case of ST-elevation myocardial infarction (STEMI) in an HIV-positive patient in whom revascularization was not possible due to anatomical complications of HIV, namely, the extent and size of coronary aneurysms.

## Case presentation

**Figure 1:**
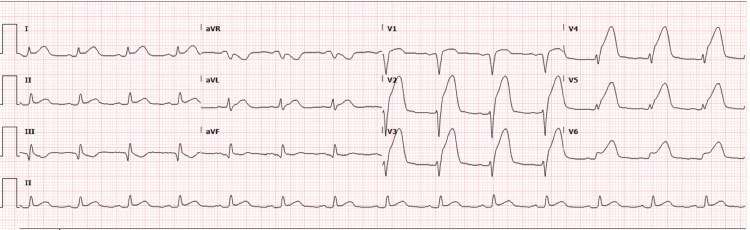
Initial EKG showing anterolateral ST segment elevations prior to cardiac catherization

**Figure 2:**
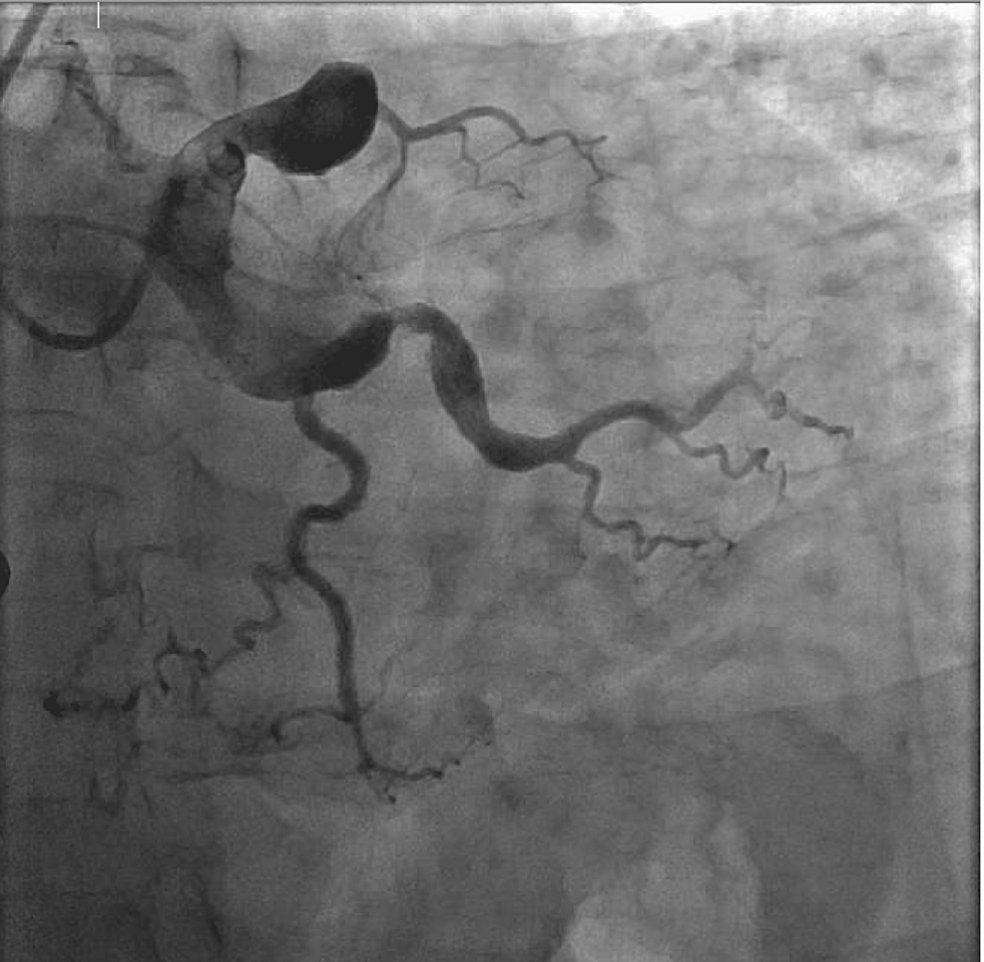
Left anterior descending coronary artery with a large proximal aneurysmal segment which is 100% occluded at the region of the first septal perforator

**Figure 3:**
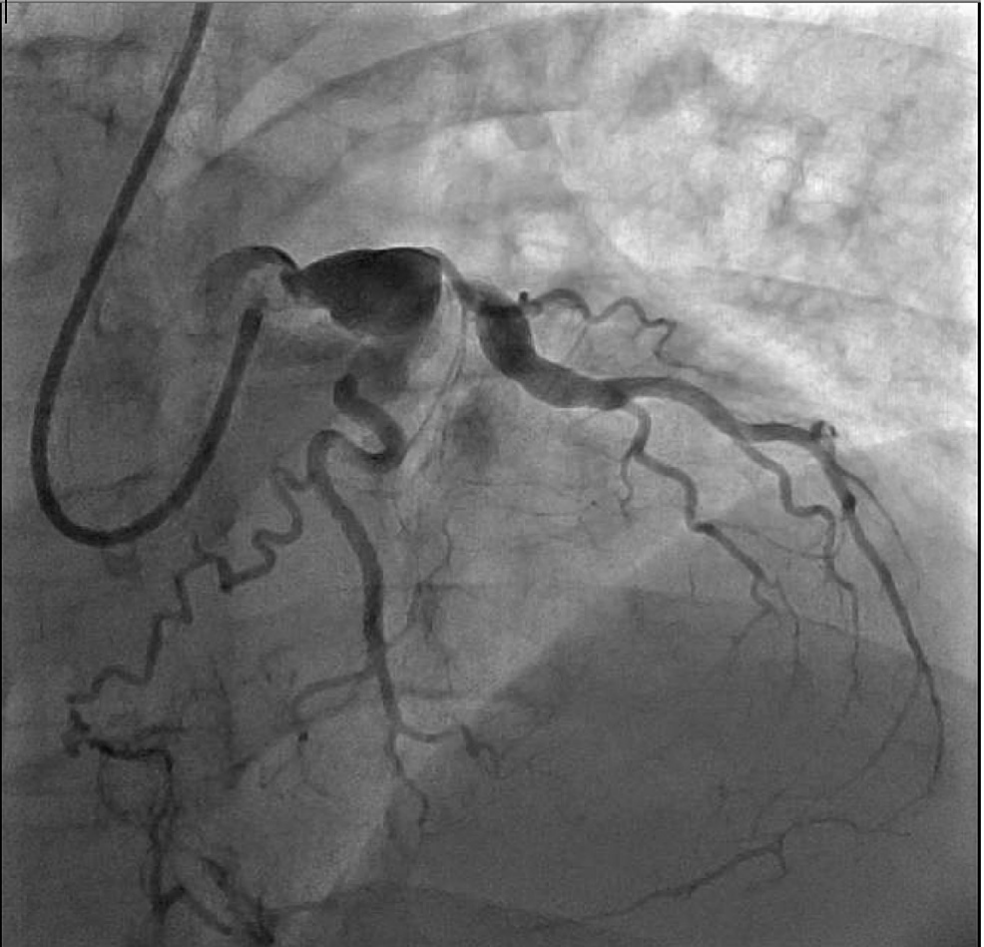
Left circumflex artery, which is a large caliber vessel with large aneurysmal segment in the proximal vessel, has large caliber obtuse marginal artery (OM1) branch that has aneurysmal dilatation in the proximal and mid segments

Our patient is a 54-year-old male with a past medical history of HIV diagnosed in 2018, moderate coronary artery disease (CAD), and paroxysmal atrial fibrillation on Eliquis, who presented to the emergency department with chest pain suggestive of an acute coronary syndrome. The patient reported waking up the morning of admission with a 6/10 in intensity non-radiating chest pain before calling Emergency Medical Services (EMS). During this time, he denied shortness of breath, nausea, vomiting, diarrhea, fevers, chills, or any other acute symptoms. En route to the hospital, an EKG was obtained (Figure 1) and showed ST elevations in leads V2-V5. In the emergency department, the patient was given aspirin, clopidogrel, high-intensity statin, and IV heparin and was emergently transported to the cardiac catheterization lab. The patient’s left heart catheterization was significant for the left main coronary artery being mildly aneurysmal with no angiographic stenosis, the left anterior descending coronary artery had a large proximal aneurysmal segment that was 100% occluded at the region of the first septal perforator, and the left circumflex coronary artery had a large aneurysmal segment in the proximal vessel. The large caliber first obtuse marginal had an aneurysmal dilatation in the proximal and mid segments. No right coronary artery images were taken given anterior EKG changes and well-developed left-to-right collaterals to fill the right posterior descending artery (PDA) and the right posterior lateral artery (PLA) (Figures 2, 3). No stents were placed at that time due to his complex anatomy and unlikely successful revascularization. The cardiothoracic surgical team was consulted as part of the multidisciplinary heart team to discuss the possibility of a coronary artery bypass graft (CABG). After a thorough discussion with the heart team, the patient was deemed not a surgical candidate given unfavorable distal bypass graft insertions along with patient preference, so the decision was made to treat him medically. Given that the patient was hemodynamically stable while on the Intra-aortic balloon pump due to left ventricular ejection fraction of 20-25%, the cardiac critical care team opted for conservative management.

**Figure 4:**
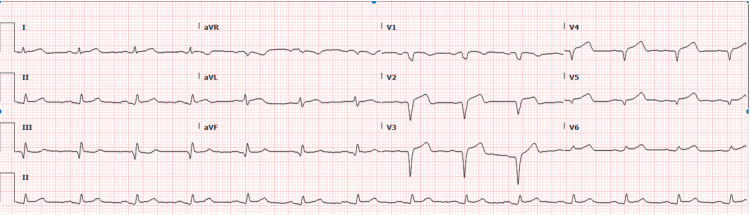
EKG post STEMI showing resolution of ST elevation in leads V2-V5; this was taken a day after the initial EKG STEMI: ST-elevation myocardial infarction

The following day, the patient remained hemodynamically stable and the intra-aortic balloon pump was removed. During removal, the patient developed an iatrogenic hematoma and eventually a pseudoaneurysm on ultrasound at the distal external iliac artery. Vascular surgery was consulted and injected the patient with thrombin at the site of the pseudoaneurysm and the patient was subsequently cleared for discharge from a vascular surgery standpoint with instructions for a repeat ultrasound the following day. The same day, the patient was transferred from the cardiac critical care unit (CCU) team to the general cardiology service. The following day, the patient underwent a repeat lower extremity arterial exam demonstrating a stable thrombosed pseudoaneurysm. The patient remained hemodynamically stable during his hospital course and remained on goal-directed medical therapy for his STEMI, which included high-dose atorvastatin, lisinopril, metoprolol succinate, clopidogrel, aspirin, and heparin drip. Repeat EKG (Figure 4) showed resolving ST elevations in V2-V5 compared to initial EKG and the patient was subsequently discharged home with close cardiology follow-up and instructed to continue Eliquis 5 mg twice daily and clopidogrel 75 mg daily and follow-up in the cardiology clinic one week post hospital discharge.

## Discussion

It is not uncommon for patients with HIV infection to present earlier in life with signs and symptoms of acute coronary syndrome given the aforementioned reasons for premature CAD. On average, an HIV-positive patient presents roughly 10 years younger than the average individual without HIV [5]. While in the general population, atherosclerosis accounts for 90% of coronary artery aneurysms, in HIV-positive patients other etiologies contribute to premature CAD including substance use disorder, infections, pro-inflammatory changes, trauma, and various connective tissue disorders [1]. Statistics show that the likelihood of developing CAD in the general population is nearly 7.2%, with about 20.1 million adults aged 20 and older having CAD. When adjusting for HIV-infected persons, this risk has a 1.5-fold to twofold increase. The reason for coronary aneurysms and atherosclerosis in HIV-positive patients stems from three main mechanisms and the vasculopathy associated with: inflammation and endothelial injury, smooth muscle cell proliferation and migration, and molecular mimicry [7]. Inflammation and endothelial cell dysfunction begin with an increase in inflammatory mediators such as interleukins and tumor necrosis factor-alpha, increased oxidative stress, and a decrease in CD4 count all of which lead to an increase in inflammation and immune dysfunction [8-9]. The inflammatory and endothelial changes lead to dyslipidemia, thrombosis, and endothelial dysfunction, all of which increase the risk of atherosclerosis and coronary aneurysms [9-10].

Smooth muscle cell proliferation and migration occur as the muscularis media possess surface receptors facilitating the entry of the HIV virus into host cells, which would lead to infiltration, disorder, and thinning of the medial layer. The entry of the viral envelope protein also has been reported to activate tissue factor 2, which is a potent pro-coagulant inducing thrombosis and ligand production thought to be instrumental in promoting atherogenesis [9]. Molecular mimicry is less understood but may play a part in aneurysm formation as it is theorized that the virus and its toxic by-products share ligands that are characterized by DNA sequence similarities to the viral envelope glycoprotein gp41 and gp120. This may result in autoimmune-mediated cell damage during infection and direct viral invasion of the aortic fibroblasts at the level of the adventitia [9]. Furthermore, external causes can also lead to the propagation of premature CAD and aneurysmal formation in HIV-positive patients given comorbidities associated with HIV with the aforementioned conditions exacerbated by daily habits including smoking and illicit drug use [11-13].

The management of coronary artery aneurysms in HIV patients is not well-defined due to its relative scarcity; however, treatment options generally include CABG, percutaneous coronary intervention (PCI) with covered stents, aneurysm ligation, resection, or marsupialization with interposition graft, and/or medical management with blood pressure control and anti-platelet therapy [3]. In our patient, the presence of coronary aneurysms made his case a diagnostic and therapeutic challenge in the setting of a STEMI. Our patient was not amenable to PCI due to his unique anatomy and unfavorable bypass graft insertion sites and was also not a candidate for CABG due to his high-risk features as he was taking clopidogrel and apixaban at home and the complexity of the aneurysms.

## Conclusions

Currently, while there are similar case reports of HIV patients with coronary aneurysms who were diagnosed with STEMI and treated medically, there are no clear guidelines on the management of coronary aneurysms in HIV-infected patients. The best understanding and medical management in HIV-positive patients with coronary aneurysms is to treat the underlying cause, HIV, using antiretrovirals. Since the life expectancy of HIV-positive patients is now dramatically improved, it is likely that the incidence of CAD in addition to its complications (such as coronary aneurysms) will also increase. With this, diagnostic and therapeutic challenges will be present and as further investigation into the pathogenesis of atherosclerosis in HIV positive patients and aneurysm formation makes advances in the upcoming years and decades, a clearer picture and treatment/management strategy may be on the horizon.

## References

[1] Heizer J, Petersen TC, Flemmer MC (2016). Multiple coronary aneurysms in a young adult with acquired immunodeficiency syndrome. Oxf Med Case Reports.

[2] Boyer N, Gupta R, Schevchuck A (2014). Coronary artery aneurysms in acute coronary syndrome: case series, review, and proposed management strategy. J Invasive Cardiol.

[3] Kashyap A, Abramov D, Bharadwaj A, Rabkin M, Rabkin DG (2022). Coronary artery aneurysm, ectasia and stenosis in a 53-year-old man with HIV infection. J Surg Case Rep.

[4] Zwoliński R, Kamerys J, Jabłonowska E, Marcinkiewicz A, Jaszewski R, Kręcki R, Jegier B (2017). Progression of coronary artery disease in a HIV-infected patient previously treated for ascending aorta aneurysm. Kardiochir Torakochirurgia Pol.

[5] Triant VA, Lee H, Hadigan C, Grinspoon SK (2007). Increased acute myocardial infarction rates and cardiovascular risk factors among patients with human immunodeficiency virus disease. J Clin Endocrinol Metab.

[6] Ayers J, Mandell R, Sanghvi K, Aboujaoude R, Hsi DH (2014). Acute coronary thrombosis and multiple coronary aneurysms in a 22-year-old man with the human immunodeficiency virus. Tex Heart Inst J.

[7] Hiremath Hiremath, P. G., Martin Martin, S. S., Blumenthal Blumenthal, R. S. (2023). Evidence-Based Review of Statin Use in Patients With HIV on Antiretroviral Therapy. https://www.acc.org/latest-in-cardiology/articles/2018/09/06/13/02/evidence-based-review-of-statin-use-in-patients-with-hiv-on-antiretroviral-therapy.

[8] Chastain DB, Henderson H, Stover KR (2015). Epidemiology and management of antiretroviral-associated cardiovascular disease. Open AIDS J.

[9] Meel R, Gonçalves R (2019). Human immunodeficiency virus associated large artery disease. Aortic Aneurysm and Aortic Dissection.

[10] Pillay B, Ramdial PK, Naidoo DP (2015). HIV-associated large-vessel vasculopathy: a review of the current and emerging clinicopathological spectrum in vascular surgical practice. Cardiovasc J Afr.

[11] Drozd DR, Kitahata MM, Althoff KN (2017). Increased risk of myocardial infarction in HIV-infected individuals in North America compared with the general population. J Acquir Immune Defic Syndr.

[12] Boccara F, Mary-Krause M, Teiger E (2011). Acute coronary syndrome in human immunodeficiency virus-infected patients: characteristics and 1 year prognosis. Eur Heart J.

[13] Rickerts V, Brodt H, Staszewski S, Stille W (2000). Incidence of myocardial infarctions in HIV-infected patients between 1983 and 1998: the Frankfurt HIV-cohort study. Eur J Med Res.

